# Does population aging promote embeddedness in the regional medical device trade network? Evidence from RCEP member countries

**DOI:** 10.3389/fpubh.2026.1969842

**Published:** 2026-10-27

**Authors:** Xinyi Li, Leran Ge, Wen Liu

**Affiliations:** 1 School of Business, Shandong University, Weihai, China; 2 School of Economics, Shandong University, Jinan, China

**Keywords:** medical device trade, population aging, public health, RCEP, trade network embeddedness

## Abstract

**Introduction:**

Enhancing member countries' embeddedness in the RCEP medical device trade network is an important issue for addressing population-aging challenges across the region and promoting coordinated development and cooperation in the healthcare industry.

**Method:**

Using data on 15 RCEP member countries from 2004 to 2023, this study constructs a regional medical device trade network and a network embeddedness index to examine how population aging affects member countries' embeddedness.

**Results:**

The results indicate that the RCEP medical device trade network exhibits high density, strong connectivity, and low centralization, with trade linkages among members continuing to deepen. Population aging significantly increases members' embeddedness in the network. Mechanism analyses reveal that population aging promotes embeddedness primarily by enhancing the specialization of export products and consolidating import sources. Heterogeneity analyses show that this positive effect is more pronounced among non-ASEAN members, developing countries, and economies at advanced stages of population aging.

**Discussion:**

These findings provide important policy insights for RCEP member countries seeking to respond to population aging, strengthen public health security capacity, and advance trade cooperation in the health and medical device sectors.

## 1 Introduction

Population aging is continuously reshaping the global structure of healthcare demand. The World Health Organization projects that the share of the global population aged 60 and over will rise from 12% in 2015 to 22% by 2050, with the population in this age group expanding from 1 billion in 2019 to 1.4 billion by 2030 and 2.1 billion by 2050. The growing older population not only drives up aggregate demand for medical services but also sustains increasing demand for diagnostic, therapeutic, rehabilitative, and long-term care medical devices (1, 2). The medical device industry combines attributes of health security, technology intensity, and advanced manufacturing, bearing on both population health and healthcare accessibility while also reflecting a country's capabilities in high-end manufacturing and public health supply. Consequently, trade in medical devices has become an increasingly important channel for countries to meet health needs, optimize medical resource allocation, and support the continuity of public health supply chains.

Meanwhile, rising uncertainty in global industrial and supply chains has drawn widespread attention to the cross-border flows and regional collaboration of critical health products such as medical devices. The COVID-19 pandemic exposed vulnerabilities in the global value chain for medical supplies regarding supply concentration, cross-border coordination, and emergency response capacity, rendering regional cooperation an important institutional arrangement for balancing supply efficiency and supply chain resilience (3, 4). Countries typically rely on multilateral or bilateral free trade agreements to reduce trade costs, stabilize economic and trade ties, and enhance the resilience of industrial and supply chains. Since entering into force on January 1, 2022, the Regional Comprehensive Economic Partnership (RCEP) has provided an institutional foundation for the flow of medical devices and industrial collaboration within the region through tariff concessions on goods trade, cumulative rules of origin, and customs procedure facilitation. However, RCEP member states differ markedly in population aging, healthcare demand structure, industrial base, and institutional environment: members with higher aging intensity exhibit stronger demand for high-end diagnostic and therapeutic equipment, chronic disease management devices, and long-term care products, whereas developing members place greater emphasis on expanding medical infrastructure and improving basic healthcare service capacity. Consequently, medical device trade in the RCEP region is not merely a matter of scale expansion, but rather a networked evolution driven by the interplay of demographic demand shifts, industrial division adjustments, and regional institutional coordination.

Against this backdrop, examining medical device trade solely through the lens of trade volume, product competitiveness, or bilateral trade relations fails to reveal member states' structural positions within the regional health product supply system and their dynamic evolution. Trade network analysis captures cross-country trade linkages, node positions, and tie strength simultaneously, offering an appropriate framework for identifying the structural effects of population aging on reshaping the regional medical device trade landscape. Existing studies show that the global medical device trade network has witnessed a continuous rise in participating entities, linkage density, and local clustering, with significant heterogeneity across economies in network influence and functional positioning (5, 6). Accordingly, this study addresses three questions: What evolutionary characteristics define the RCEP regional medical device trade network? Does population aging significantly affect member states' degree of trade network embeddedness? Through what mechanisms does this effect operate? By taking demand-side population aging as the point of departure, this study explains the evolution of the regional medical device trade network, with a view to providing empirical evidence for RCEP members to deepen industrial division in the health sector, optimize medical device supply allocation, and inform resilience-oriented regional cooperation.

Related studies can be broadly grouped into two strands. The first examines population aging and healthcare demand. Population aging exerts long-term effects on macroeconomic performance by altering labor supply, savings structure, fiscal expenditure, and consumption demand (7, 8). As individuals age, their stock of health capital depreciates more rapidly, increasing the risk of chronic disease and the need for rehabilitation, nursing, and long-term care, thereby expanding demand for health services (9). Healthcare expenditure among older adults is both high and growing rapidly, which further drives up demand for healthcare services (10). Income growth also shifts the optimal expenditure structure toward health, as the marginal utility of general consumption declines rapidly, whereas the marginal utility of additional life expectancy does not decline proportionally (11). The effects of aging on healthcare expenditure are reflected not only in its scale, but also in changes in health status, patterns of service utilization, and the structure of technology adoption (1, 12). With respect to the drivers of healthcare expenditure growth, the age-effect perspective argues that per capita healthcare expenditure is higher among individuals aged 65 and above than among other age groups; accordingly, age is positively associated with total healthcare expenditure (13). Building on this foundation, Buchner and Wasem (14) proposed the Steepening Hypothesis. By contrast, the Red Herring Hypothesis emphasizes that healthcare expenditure is driven primarily by proximity to death rather than chronological age itself (15). Kallestrup-Lamb et al. (16) find that both effects can simultaneously contribute to healthcare expenditure growth, with the steepening effect playing the dominant role, and that the two effects interact to reinforce one another among older populations. Recent research further shows that population aging can reshape growth and productivity incentives through changes in labor supply, automation, and productivity dynamics (17, 18). Evidence on health spending also indicates that demographic aging changes expenditure profiles over time, although the magnitude depends on health status and proximity to death (19).

The second strand of research concerns regional economic integration and the evolution of trade networks. Traditional gravity models suggest that factors such as economic size, geographic distance, common language, shared borders, and trade agreements significantly shape bilateral trade flows (20). Within this framework, the RCEP's tariff concessions, harmonized rules of origin, customs facilitation, and promotion of regional supply chain integration provide an institutional basis for member states to deepen trade ties, strengthen regional trade linkages, and advance the integration of production networks in East Asia (21). From a network perspective, regional economic integration is reflected in changes in network density, node centrality, local clustering, and cross-country interdependence. Kali and Reyes (22) were among the first to introduce network analysis into the study of international economic integration, arguing that a country's position in a trade network reflects its degree of economic integration. In the case of differentiated products, international trade depends more heavily on social networks and relational ties, through which trading partners build trust and share information, thereby reducing search costs (23). Related studies of the world trade network document persistent topological structure and evolving cross-country connectivity (24, 25), while broader network theory provides a basis for interpreting centrality, local closure, and structural position (26).

A growing recent literature directly connects demographic change with international trade and long-run structural transformation. Brakman et al. (27) use a structural gravity framework to show that changes in the size and age composition of national labor supply can substantially reshape the geographical distribution of world trade over the twenty-first century. Bekkers et al. (28) incorporate demographic projections into a recursive dynamic general-equilibrium model and show that future trade patterns emerge from the interaction of demographic, technological, sectoral, and trade-cost changes. Liu et al. (29) likewise treat demographic change through labor-supply growth as a core driver of structural transformation in a multi-region, multi-sector global model, with its effects transmitted through international trade and intersectoral linkages. These studies reinforce the view that demographic structure can influence not only domestic demand and factor supply but also the scale, composition, and spatial configuration of international trade. However, how population aging affects a country's embeddedness in sector-specific trade networks, particularly in health-related industries, remains insufficiently understood.

In summary, although existing studies have examined population aging and the evolution of regional trade networks, thereby providing an important foundation for this study, several gaps remain. First, research on population aging has focused primarily on healthcare expenditure and demand for health services, with limited attention to cross-border trade in medical devices and its implications for network structure. Second, studies of medical device trade have mainly examined global network characteristics and the identification of core nodes, but have not fully explained how demographic change affects a country's degree of trade network embeddedness. Third, research on RCEP trade networks has rarely incorporated population aging, the division of labor in the health industry, and regional network embeddedness into a unified analytical framework. Against this background, this study uses a sample of RCEP member states to investigate the effect of population aging on their degree of embeddedness in medical device trade networks.

The marginal contributions of this study are 3-fold. First, it extends the literature on the economic effects of population aging. Rather than focusing only on conventional dimensions such as labor force changes and economic growth, this study links population aging to trade and shifts the analytical focus toward regional trade networks. Specifically, it constructs an index of trade network embeddedness based on two dimensions, namely network accessibility and local relational closure, and uses this index to systematically examine how population aging affects the structural position of RCEP member states in the medical device trade network. In doing so, it shows that demographic change not only alters the demand for and scale of medical device trade, but also reshapes the degree to which member states are embedded in the regional health product supply system. Second, this study proposes and tests a new mechanism through which population aging promotes embeddedness in medical device trade networks. It empirically examines this mechanism from two perspectives, export supply adjustment and import sourcing restructuring, and identifies two transmission channels: the specialization of export products, and the consolidation of import sources. This analysis helps uncover the underlying logic through which population aging affects the structure of medical device trade networks, and provides empirical evidence for understanding the deepening division of labor in the regional health industry, the stabilization of trade relationships, and the restructuring of supply chains in aging societies. Third, this study enriches the literature on heterogeneity in the relationship between population aging and medical device trade. It conducts heterogeneity analysis from three dimensions, namely regional characteristics, level of economic development, and stage of population aging, to systematically examine the differential effects of population aging on trade network embeddedness across ASEAN and non-ASEAN members, developing and high-income economies, and economies at different stages of aging. In addition to broadening the depth and scope of the relevant literature, this study provides more targeted empirical evidence to support RCEP member states in formulating differentiated, aging-responsive policies for the medical device industry, trade cooperation, and supply chain security.

## 2 Theoretical framework and research hypotheses

According to health capital theory, aging accelerates the depreciation of individual health capital, increases demand for healthcare services and the use of medical devices, and makes such demand highly persistent and relatively inelastic (9). Population aging not only expands the scale of demand, but may also reshape the structure of bilateral trade relationships (30). On the one hand, healthcare demand induced by population aging is inherently persistent (2), which may increase the likelihood of sustained trade between importing and supplying countries. On the other hand, medical devices are typically accompanied by specialized services, including equipment maintenance, consumables supply, technical training, quality certification, and after-sales support, which encourages trading partners to form more stable cooperative relationships (31). Therefore, population aging may contribute to the continued deepening of medical device trade relationships by increasing transaction frequency, enhancing relationship stability, and strengthening relationship-specific investments.

In trade networks, sustained and stable trade relationships can shorten the effective trade distance between countries (32). From the export side, changes in consumer demand induced by population aging create incentives to adjust production and supply structures (33). From the import side, steadily growing demand can increase the economic value of existing trade relationships, thereby reinforcing supplier selection mechanisms based on reputation and relational contracts (34). The sustained matching of supply and demand not only strengthens a country's tie strength with its core trading partners, but also increases local clustering through shared suppliers, common import markets, and after-sales service networks. Therefore, population aging can enhance a country's degree of embeddedness in regional medical device trade networks by expanding product demand, stabilizing trade relationships, and deepening cooperative ties. Based on this, this study proposes the following hypothesis:

Hypothesis 1. Population aging significantly increases the degree of embeddedness in regional medical device trade networks.

Population aging may promote the specialization of medical device exports by altering both factor supply and market demand. As the old-age dependency ratio rises and labor becomes relatively scarce, firms face tighter cost constraints and stronger incentives to adopt technological substitutes, making them more likely to undertake strategic transformation (35) and reallocate resources toward higher-performing products (36). At the same time, the sustained growth in healthcare demand driven by population aging expands the market potential for specific products, prompting firms to specialize in areas such as chronic disease diagnosis and treatment, rehabilitation and nursing, health monitoring, and long-term care. Given the high fixed costs associated with medical device research and development, registration and certification, and after-sales service, export-oriented firms operating across too many product categories may face diseconomies of scope (37). By contrast, a moderate increase in product concentration can help generate economies of scale, build quality reputation, and accumulate technological capabilities, thereby shifting the export structure toward more competitive products.

Specialization in export products may further affect medical device trade network embeddedness. Specialized production helps exporting countries improve supply stability and quality consistency in specific product segments, thereby strengthening trade intensity and the durability of their relationships with importing markets. For economies with technological advantages, such specialization is mainly reflected in a focus on high-end diagnostic and therapeutic equipment, key components, and precision instruments. For manufacturing-based economies, it may take the form of large-scale supply of low- to mid-range devices, medical consumables, and supporting products (38). As exporting countries strengthen their supply capacity in products in which they hold a competitive advantage, transaction frequency, the stability of cooperation, and ties with common trading partners all increase, thereby promoting local network closure and enhancing the degree of trade network embeddedness. This leads to the following hypothesis:

Hypothesis 2. Population aging promotes trade network embeddedness by increasing the degree of specialization in medical device exports.

Population aging may promote the consolidation of import sources by altering procurement behavior in medical device markets. As demand for age-related diagnosis, treatment, rehabilitation, monitoring, and long-term care continues to grow, procuring entities place greater emphasis on product quality, registration and certification, supply stability, and after-sales service capacity. Medical devices are highly technical and exhibit substantial variation in product characteristics, so trade networks can play an important role in reducing search costs (23). At the same time, quality risks, regulatory barriers, and safety requirements make importers more likely to select suppliers with recognized certification, consistent quality, and well-established service systems. High-quality suppliers reduce the probability that importers will switch trading partners, thereby fostering long-term and stable trade relationships (39). Consequently, population aging may shift procurement patterns away from extensive search and toward more concentrated relationships with a limited number of reliable suppliers, thereby reducing compliance, information, and switching costs while improving the continuity and security of medical device supply. This supplier-selection mechanism is especially relevant in medical-device markets, where quality uncertainty and regulatory oversight shape market access and procurement decisions (40).

The consolidation of import sources may further affect medical device trade network embeddedness. Import source consolidation implies that trade relationships may shift from dispersed, small-scale, and sporadic transactions toward stable, long-term, and high-frequency transactions. Stable supply relationships are more conducive to relationship-specific investment, thereby increasing trade intensity and the stability of trade relationships between importing countries and core supplier countries. As core supply relationships become more stable, the effective trade distance between countries declines and ties among common trading partners become tighter, thereby increasing the degree of embeddedness in medical device trade networks. This leads to the following hypothesis:

Hypothesis 3. Population aging increases trade network embeddedness by promoting the consolidation of medical device import sources.

## 3 Research design

### 3.1 Sample selection and data sources

This study examines the effect of population aging on regional medical device trade network embeddedness using the 15 RCEP member states over the sample period 2004 to 2023. The sample therefore captures the long-run evolution of trade among the RCEP countries. The 15 RCEP members included in the sample are Australia, Brunei Darussalam, Cambodia, China, Indonesia, Japan, Lao PDR, Malaysia, Myanmar, New Zealand, the Philippines, Singapore, South Korea, Thailand, and Vietnam.

The sample begins in 2004 mainly because this starting point ensures the continuity of medical device trade data, the availability of product codes, and the relative stability of regional trade linkages. In addition, trade ties in the Asia-Pacific region have continued to deepen since China's accession to the WTO, making this period a relatively complete time window for examining the long-run evolution of the regional medical device trade network. Medical device trade data are obtained from the UN Comtrade database. Bilateral trade values are measured in current U.S. dollars; year fixed effects absorb common nominal price and exchange-rate shocks over time. Following the 2021 revised Regulations on the Supervision and Administration of Medical Devices, this study defines medical devices and identifies 117 product categories based on six-digit HS codes. These products are further grouped into 10 categories along the industrial chain. The upstream segment includes two categories: core raw materials and electronic and mechanical components. The midstream segment includes five categories: diagnostic equipment, therapeutic equipment, high-value consumables, low-value consumables, and *in vitro* diagnostics. The downstream segment includes three categories: home-use medical devices, rehabilitation assistive devices, and distribution-related support products. Data on population structure are drawn from the World Bank's World Development Indicators database. This study measures the degree of population aging using the old-age dependency ratio, defined as the ratio of the population aged 65 and above to the population aged 15–64. The control variables are obtained from the World Bank WDI, CEPII BACI, and Worldwide Governance Indicators (WGI) databases. GDP per capita and total population are transformed into natural logarithms, while ratio, percentage, and index variables are retained in their original units.

### 3.2 Construction of the medical device trade network and related measures

This study constructs an annual directed and weighted medical device trade network by treating RCEP member states as nodes and the trade relationships among them as directed edges. In year t, if country i exports medical devices to country j, a trade link is established from i to j. The corresponding element in the adjacency matrix takes the value of 1 when the bilateral trade value exceeds the baseline threshold of USD 500,000, and 0 otherwise. The threshold is expressed in current U.S. dollars and held constant across years. Bilateral trade values are further used to measure edge weights, and self-loops are excluded.

Let the RCEP medical device trade network be defined as:


$$\begin{array}{l} G\left(t\right)=\left(V,E_{t},W_{t}\right) \end{array}$$ (1)

Here, *V* denotes the set of nodes representing RCEP member states, *E*_*t*_ denotes the set of trade links in year *t*, and *W*_*t*_ denotes the corresponding edge-weight matrix. The trade matrix can be further expressed as follows:


$$A_{ijt}=\{\begin{array}{c} 1,Trade_{ijt}>500,000\\ 0,Trade_{ijt}\leq 500,000 \end{array}$$ (2)

where *A*_*ijt*_ is an indicator variable equal to 1 if there is a medical device trade link from exporting country i to importing country j in year *t*, and 0 otherwise, while *Trade*_*ijt*_ denotes the corresponding export value. To mitigate the influence of extreme trade values, edge weights are defined only for links that pass the threshold, as *W*_*ijt*_ = *A*_*ijt*_·*ln*(*Trade*_*ijt*_+1), which captures the intensity of trade links. Thus, flows above USD 500,000 define both a binary link and a positive edge weight; flows at or below the cutoff are coded as zero in both the adjacency matrix and the weight matrix.

Throughout Equations 1–5, iandj index exporting and importing countries, respectively, t indexes year, and N is the number of member countries; self-loops are excluded. The directed matrix is retained for in-degree, out-degree, weighted in-degree, and weighted out-degree. To apply the Onnela et al. (41) weighted-clustering formulation consistently, bilateral weights are symmetrized as $W_{ijt}^{s}=W_{ijt}+W_{jit}$ before weighted closeness centrality and the weighted clustering coefficient are calculated. These measures therefore capture overall bilateral accessibility and local relational closure.

#### 3.2.1 Overall network measures

The overall evolution of the RCEP medical device trade network is characterized using network density, average degree, weighted degree, network centralization, and modularity. Network density measures the share of actual trade links among member states in all possible trade links, and is calculated as follows:


$$\begin{array}{l} Density_{t}=\frac{\underset{i}{\sum }\underset{j}{\sum }A_{ijt}}{N\left(N-1\right)} \end{array}$$ (3)

*N* denotes the number of RCEP member states. A higher value of network density indicates more extensive medical device trade linkages within the region. Average degree reflects the average number of trading partners per member state, while weighted degree further incorporates information on trade volumes. Network centralization captures the extent to which the network is dominated by a small number of nodes, whereas modularity reflects the clarity of community structure within the network.

#### 3.2.2 Node-level measures and trade network embeddedness

At the node level, in-degree, out-degree, weighted in-degree, weighted out-degree, betweenness centrality, and eigenvector centrality are calculated to describe the number of trading partners, trade scale, intermediary role, and the extent of each member state's connection with core nodes. The weighted in-degree and weighted out-degree measures are transformed using natural logarithms. These indicators are primarily used to characterize the structural features of the regional network, whereas the empirical analysis focuses on trade network embeddedness, which captures network accessibility and the degree of local relational closure. Eigenvector centrality is interpreted following Bonacich (42), so connections to more central trading partners contribute more strongly to a country's centrality score.

Trade network embeddedness is measured along two dimensions: weighted closeness centrality and the weighted clustering coefficient. The former reflects the average effective distance from a country to all other nodes, while the latter measures the degree of closure among its trading partners. Taken together, these two dimensions capture both a country's accessibility within the regional network and the closeness of its local trade relationships.

First, weighted closeness centrality. Following Freeman's (43) standardization method and Newman's (44) extension to weighted networks, network distance is constructed as the reciprocal of trade link strength, and the shortest weighted paths from a given country to all other member states are then calculated accordingly. A higher value of weighted closeness centrality indicates shorter effective trade distances between a country and other nodes in the region, and thus stronger network accessibility. The measure is calculated as follows:


$$\begin{array}{l} C_{clo}^{W}\left(i,t\right)=\frac{N-1}{\underset{j\neq i}{\sum }d_{t}^{W}\left(i,j\right)} \end{array}$$ (4)

where $d_{t}^{W}\left(i,j\right)\;$denotes the weighted shortest-path distance calculated using Dijkstra's algorithm.

Second, the weighted clustering coefficient. Following Onnela et al. (41), the weighted clustering coefficient is defined as the geometric-mean intensity of the closed triangles around a node, where edge weights are first normalized by the maximum edge weight in the network:


$$\begin{array}{l} C_{clu}^{W}\left(i,t\right)=\frac{2}{k_{it}\left(k_{it}-1\right)}\underset{j<h}{\sum }{\left({ŵ}_{ijt}^{s}\cdot {ŵ}_{iht}^{s}\cdot {ŵ}_{jht}^{s}\right)}^{\frac{1}{3}} \end{array}$$ (5)

where **k**_**it**_ denotes the number of trading partners of node i and ${\hat{w}}^{s}$ is the normalized symmetric edge weight.

### 3.3 Variable definitions

#### 3.3.1 Dependent variable

The dependent variable is a country's degree of trade network embeddedness within the region. Weighted closeness centrality and the weighted clustering coefficient are first standardized using Z-scores, and a composite index is then constructed as the equally weighted average of the two measures. The Z-score standardization is performed over the full country-year sample.

#### 3.3.2 Key explanatory variable

The core explanatory variable is the old-age dependency ratio, defined as the ratio of the population aged 65 and above to the population aged 15 to 64, multiplied by 100. Compared with the share of the population aged 65 and above in the total population, this measure captures not only the relative size of the older population in need of care, but also its relationship to the potential labor force and contribution-paying population, thereby providing a more comprehensive reflection of healthcare demand growth and supply-side constraints. In the robustness analysis, the share of the population aged 65 and above in the total population is used as an alternative measure of the core explanatory variable.

#### 3.3.3 Control variables

Following Anderson and van Wincoop (20), De Groot et al. (45), Bloom et al. (8), and Gu and Stoyanov (46), the analysis includes the following control variables: (1) GDP per capita, which captures residents' purchasing power and the level of economic development; (2) total population, which controls for potential market size and the demand base for medical devices; (3) the urbanization rate, which reflects access to healthcare services, population concentration, and logistics and distribution conditions; (4) trade openness, which measures a country's participation in the international division of labor and cross-border trade linkages; (5) net FDI inflows as a share of GDP, which reflect cross-border capital linkages and the inflow of external technology and managerial expertise; (6) the Internet penetration rate, which captures the level of digital infrastructure and the capacity for cross-border information coordination; (7) the labor force participation rate, which controls for the effects of demographic change on labor supply and economic activity; and (8) government effectiveness, which captures differences in the quality of public services and the capacity for policy formulation and implementation. In addition, country fixed effects are included to account for time-invariant country characteristics, such as geographic location, cultural background, long-term institutional environments, and industrial endowments. Year fixed effects are also included to control for time-specific shocks, including global advances in medical technology, financial crises, public health emergencies, and common price fluctuations.

#### 3.3.4 Instrumental variables

To address endogeneity arising from omitted variables and reverse causality, the analysis employs an instrumental variables approach. Specifically, the shares of the population aged 50–54 and 55–64 15 years earlier are used as two instruments for the old-age dependency ratio. For these instruments to be valid, they must satisfy both the relevance condition and the exclusion restriction. With respect to relevance, the cohorts aged 50–54 and 55–64 15 years earlier enter the 65–69 and 70–79 age groups, respectively, 15 years later and therefore constitute an important part of the current older population. Historical cohort shares should thus be strongly correlated with the current old-age dependency ratio. With respect to the exclusion restriction, population cohort structure 15 years earlier was determined mainly by historical fertility, mortality, and migration patterns, and is unlikely to directly affect a country's current structural position in the medical device trade network. After controlling for country fixed effects, year fixed effects, population size, economic development, and other relevant factors, historical cohort structure is expected to affect medical device trade network embeddedness primarily through its impact on the current level of population aging, rather than through any systematic correlation with the error term. This supports the plausibility of the exclusion restriction.

#### 3.3.5 Mediating variables

To further identify the transmission channels through which population aging affects medical device trade network embeddedness, this study constructs two mediating variables along the dimensions of export product specialization and import source consolidation. Following Baron and Kenny (47), the mediating effects are tested using a stepwise regression approach. Specifically, (1) Export product specialization, measured by the natural logarithm of the number of medical device export product categories, with lower values indicating a narrower export product range and a higher degree of specialization; (2) Import source consolidation, measured by the number of global source countries from which each RCEP member imports medical devices, with lower values indicating a more concentrated import-source structure.

### 3.4 Model specifications

To examine the effect of population aging on medical device trade network embeddedness among RCEP member states, the following two-way fixed-effects baseline model is specified:


$$\begin{array}{l} Embed_{it}=\alpha +\beta Aging_{it}+\gamma X_{it}+\mu_{i}+\lambda_{t}+\varepsilon_{it} \end{array}$$ (6)

In Equation 6, *Embed*_*it*_ denotes the degree of medical device trade network embeddedness for RCEP member state i in year *t*; *Aging*_*it*_ denotes the level of population aging; *X*_*it*_ is a vector of time-varying control variables; μ_*i*_ denotes country fixed effects; λ_*t*_ denotes year fixed effects; and ε_*it*_ is the error term. The coefficient of primary interest is β. A significantly positive estimate of β indicates that population aging increases the embeddedness of member states in the regional medical device trade network.

To further examine the transmission mechanisms through which population aging affects regional medical device trade network embeddedness, the analysis follows Baron and Kenny (47) and tests for mediating effects using a stepwise regression approach. Two additional models are specified on the basis of Equation 6 as follows:


$$\begin{array}{l} M_{it}=\alpha_{1}+\beta_{1}Aging_{it}+\gamma_{1}X_{it}+\eta_{i}+\lambda_{t}+\varepsilon_{it} \end{array}$$ (7)

$$\begin{array}{l} Embed_{it}=\alpha_{2}+\beta_{2}Aging_{it}+\theta M_{it}+\gamma_{2}X_{it}+\eta_{i}+\lambda_{t}+\varepsilon_{it} \end{array}$$ (8)

In Equations 6 and 7, *M*_*it*_ denotes the mediating variable. A mediating effect is supported if both the coefficient β_1_ on *Aging*_*it*_ and the coefficient θ on *M*_*it*_ are statistically significant. The remaining variables are defined as in the baseline regression model. Equation 7 is estimated separately for the two mediating variables. For the export-product channel, Mit is quantified as the natural logarithm of the number of medical device export product categories; a lower value indicates greater export-product specialization. For the import-source channel, *M*_*it*_ is quantified as the number of countries worldwide from which each RCEP member imports medical devices; a lower value indicates greater consolidation of import sources. Thus, *M*_*it*_ represents one mediator at a time rather than a single composite measure. Equation 8 involves incorporating the mediating variable into the base regression equation to test the influence of the explanatory variable on the dependent variable through *M*_*it*_.

Table 1 reports the descriptive statistics for the main variables. Trade network embeddedness, measured as an equally weighted composite index of standardized weighted closeness centrality and the weighted clustering coefficient, has a mean close to zero and a standard deviation of 0.970, indicating substantial variation across member states and over time. The old-age dependency ratio has a mean of 13.625, a minimum of 4.314, and a maximum of 50.285, suggesting that RCEP member states span a wide spectrum from relatively young population structures to deeply aging economies.

**Table 1:** Descriptive statistics.

Category	Variable	Definition	Observations	Mean	S. D.	Min	Max
Dependent variables	Trade network embeddedness	Measures a country's degree of embeddedness in the RCEP medical device trade network, reflecting its accessibility to trade linkages with other member states and the closeness of its local trade relationships.	300	0	0.97	−1.668	3.122
	Weighted closeness centrality	Measures the effective distance from a country to other member states in the weighted trade network; a higher value indicates stronger network accessibility.	300	9.681	1.975	5.432	13.823
	Weighted clustering coefficient	Measures the degree of closure in local trade relationships among a country's trading partners; a higher value indicates tighter local network linkages.	300	0.015	0.012	0.001	0.067
Explanatory variables	Old-age dependency ratio	Defined as the ratio of the population aged 65 and above to the working-age population, and used to measure the degree of population aging.	300	13.625	9.448	4.314	50.285
	Share of population aged 65 and above	Defined as the share of the population aged 65 and above in the total population, and used as an alternative measure of population aging.	300	9.13	5.86	3.003	29.562
Control variables	GDP per capita	Measured as the natural logarithm of GDP per capita and used to capture the level of economic development.	300	9.022	1.409	6.138	11.13
	Population size	Measured as the natural logarithm of total population and used to capture market size and the population base.	300	17.3	1.883	12.774	21.069
	Urbanization rate	Defined as the share of the urban population in the total population and used to reflect the level of urbanization.	300	61.098	24.178	18.981	100
	Trade openness	Defined as the ratio of total imports and exports to GDP and used to capture a country's degree of openness to international trade.	300	91.005	57.198	21.658	294.247
	Net FDI inflows as a share of GDP	Defined as net foreign direct investment inflows as a share of GDP and used to reflect capital openness and the level of foreign capital inflows.	300	4.315	5.564	−3.601	32.596
	Internet penetration rate	Defined as the share of Internet users in the total population and used to reflect digital infrastructure and access to information.	296	53.593	31.873	0.024	98.97
	Labor force participation rate	Defined as the share of the labor force in the corresponding working-age population and used to reflect labor supply conditions.	300	66.854	5.959	54.746	85.439
	Government effectiveness	Measures the quality of public services, policy implementation capacity, and the level of institutional governance.	300	0.499	1.065	−1.724	2.293
Mediating variables	Number of export product categories	The natural logarithm of the number of medical device export product categories, reflecting the breadth of export product coverage or the degree of export product specialization.	300	4.614	0.293	3.296	4.771
	Number of import source countries	Measures the number of source countries for medical device imports and is used to reflect the degree of dispersion or consolidation of import sources.	300	122.397	43.247	33	201

## 4 The RCEP regional medical device trade network analyse

### 4.1 Overall network characteristics

The phased characteristics of the RCEP medical device trade network in 2010, 2015, 2020, and 2023 are presented in Table 2. Network density increased from 0.848 in 2010 to 0.952 in 2023, indicating that medical device trade linkages among member states have approached full connectivity. The average path length remained stable at around 1.1 throughout the period, suggesting a high degree of connectivity among nodes within the region. The average clustering coefficient rose from 0.876 to 0.960, indicating a continued increase in the degree of closure among local trade relationships. Betweenness centralization declined from 0.064 to 0.005, suggesting that the network has become less dependent on a small number of intermediary nodes and that its overall structure has become flatter.

**Table 2:** Overall network characteristics of the RCEP medical device trade network.

Year	2010	2015	2020	2023
Network density	0.848	0.895	0.933	0.952
Average clustering coefficient	0.876	0.913	0.945	0.960
Average path length	1.152	1.105	1.067	1.081
Betweenness centralization	0.064	0.014	0.003	0.005

In terms of node functions, Singapore plays a pivotal role in regional distribution and transshipment trade because of its open economy, strategic location, and logistical advantages. Because UN Comtrade does not uniformly separate re-exports across reporting countries, Singapore's centrality should be interpreted as reflecting both domestic trade and its regional distribution and transshipment functions. China, Japan, and South Korea, drawing on their market size, manufacturing capacity, and technological strengths, have developed relatively close industrial linkages with members such as Vietnam, Thailand, and Malaysia. Functional differences across member states jointly shape the multilayered structure of the RCEP medical device trade network.

### 4.2 Network community structure

The analysis uses the Louvain modularity-maximization algorithm to examine the clustering characteristics of medical device trade linkages (Table 3). The same resolution setting is applied across years, and repeated runs are used to retain the partition with the highest modularity. A higher level of modularity indicates clearer boundaries between network communities. Regional modularity within the RCEP declined from 0.011 in 2010 to 0.004 in 2023 and remained at a relatively low level throughout the period, suggesting that trade linkages among member states extend widely across potential community boundaries and that the regional network lacks a stable and clearly defined community structure. This finding is consistent with the high levels of network density and clustering, which reflect the extensive interconnectedness of trade linkages within the region.

**Table 3:** Community structure of the global and RCEP medical device trade networks.

Year	Global modularity	RCEP modularity
2010	0.284	0.011
2015	0.232	0.004
2020	0.232	0.003
2023	0.276	0.004

### 4.3 Node-level structure

Changes in the network positions of RCEP member states are examined from four perspectives: degree centrality, weighted degree, betweenness centrality, and eigenvector centrality (Table 4). In the later years of the sample period, the in-degree and out-degree of most member states approached the theoretical maximum of 14, indicating a high level of connectivity in the regional network. In terms of weighted degree, China, Japan, South Korea, and Singapore ranked highest in both imports and exports, followed by Vietnam, Thailand, Malaysia, Australia, and New Zealand. Although Cambodia, Brunei, Laos, and Myanmar had relatively small trade volumes, they had already established trade linkages with most other members.

**Table 4:** Node-level characteristics of the RCEP medical device trade network

Year	Country/region	Degree centrality		Log weighted degree		Betweenness centrality	Eigenvector centrality
		In-degree	Out-degree	In-weighted degree	Out-weighted degree		
2010	CHN	13	14	24.253	23.638	0.005	0.000
	IRN	11	14	21.097	21.549	0.002	0.062
	KOR	13	14	22.915	24.256	0.005	0.254
	TIIA	13	14	22.124	22.329	0.005	0.151
	AIS	12	14	21.770	20.095	0.006	0.116
	SCP	12	14	21.183	21.685	0.006	0.090
	MYS	12	14	22.897	22.946	0.006	0.078
	VSNM	13	14	21.096	19.635	0.005	0.075
	IDN	12	14	21.999	20.580	0.006	0.058
	PHL	11	13	22.019	19.321	0.000	0.076
	NZL	11	13	20.014	18.962	0.000	0.120
	MEX	10	1	18.253	9.738	0.000	0.005
	KHM	12	10	17.992	15.525	0.000	0.005
	LAO	12	3	17.316	11.731	0.000	0.031
	IBN	11	12	17.653	15.813	0.000	0.000
2015	CHN	14	14	24.109	24.181	0.002	0.064
	IRN	14	14	21.981	21.310	0.002	0.611
	KOR	13	14	23.290	24.334	0.006	0.254
	TIIA	13	14	22.300	22.641	0.016	0.118
	VSNM	14	14	22.611	21.721	0.002	0.139
	SCP	13	14	21.152	21.815	0.006	0.139
	AIS	13	14	21.846	20.344	0.006	0.132
	MYS	13	14	22.641	21.328	0.006	0.120
	IDN	12	14	21.731	20.461	0.002	0.075
	PHL	12	14	21.006	21.135	0.002	0.092
	NZL	12	14	20.330	19.021	0.002	0.085
	KHM	11	12	19.135	15.527	0.000	0.015
	VNM	12	7	19.386	14.922	0.000	0.012
	LAO	11	4	18.251	15.545	0.000	0.000
	IBN	11	11	18.005	15.487	0.000	0.001
2020	CHN	14	14	25.514	24.622	0.006	0.067
	IRN	14	14	21.099	21.230	0.006	0.000
	KOR	14	14	23.381	24.025	0.006	0.254
	VSNM	14	14	24.129	23.092	0.006	0.075
	AIS	14	14	22.311	20.326	0.006	0.165
	TIIA	14	14	22.657	22.338	0.006	0.142
	SCP	14	14	21.386	21.577	0.006	0.141
	MYS	13	14	21.116	21.006	0.006	0.125
	IDN	14	14	22.003	20.783	0.006	0.096
	PHL	14	13	22.067	22.056	0.006	0.064
	NZL	13	14	21.657	19.384	0.006	0.058
	KHM	11	12	20.183	17.445	0.000	0.017
	MMR	12	11	19.726	16.497	0.000	0.015
	LAO	10	9	18.547	16.330	0.000	0.002
	BRN	11	11	18.114	15.358	0.000	0.001
2023	CHN	14	14	24.536	24.234	0.011	0.649
	JPN	14	14	23.312	22.691	0.011	0.550
	KOR	14	14	23.236	24.045	0.011	0.312
	VNM	14	14	23.908	22.805	0.011	0.234
	THA	14	14	22.820	22.229	0.011	0.194
	SGP	13	14	23.332	24.129	0.005	0.159
	MYS	13	14	23.108	23.834	0.005	0.149
	AUS	13	14	22.010	20.627	0.005	0.146
	IDN	13	14	22.210	20.600	0.005	0.110
	PHL	13	13	22.013	22.415	0.007	0.067
	NZL	14	13	20.552	19.211	0.009	0.029
	KHM	11	13	19.697	17.382	0.000	0.020
	MMR	11	10	19.239	16.461	0.000	0.015
	LAO	10	7	18.789	16.363	0.000	0.004
	BRN	12	11	17.899	14.924	0.000	0.001

Differences in betweenness centrality across RCEP member states are generally small, with the maximum value in 2023 reaching only 0.011, indicating that regional trade exhibits limited dependence on any single transit hub. Eigenvector centrality shows a clear gradient distribution, with China, Japan, and South Korea forming the top tier and Vietnam displaying a particularly notable upward trend. Considered together with weighted degree, these patterns suggest that substantial differences in trade scale and functional specialization still persist within the region. Japan's eigenvector centrality has remained high over time, but its hub position in the network visualization is not consistently prominent. This suggests that Japan exerts influence primarily through stable linkages with core regional nodes rather than through transshipment trade. Given its technological advantages in high-end equipment and key components, Japan's role in the network is reflected more in its strong embeddedness in core industrial chains.

Overall, the RCEP medical device trade network exhibits a trend toward high connectivity and a flatter structure, with the expansion of trade partnerships within the region approaching saturation. Under such a structure, demand growth induced by population aging is more likely to deepen trade network embeddedness by increasing local clustering and shortening effective trade distances.

## 5 Empirical results

### 5.1 Baseline regression results

Building on the characterization of the trade network structure of RCEP member states, the analysis next turns to the baseline regressions. Table 5 reports the estimation results from two-way fixed-effects models with country and year fixed effects. Column (1) includes only country and year fixed effects; the estimated coefficient on the old-age dependency ratio is 0.0273 and is significantly positive at the 5% level. After GDP per capita, population size, and the urbanization rate are added in column (2), the coefficient increases to 0.0448 and remains significant at the 1% level. Column (3) further controls for trade openness and net FDI inflows, yielding a coefficient of 0.0484 that is still significant at the 1% level. After all control variables are included in column (4), the coefficient on the old-age dependency ratio rises to 0.0571 and remains significant at the 1% level. In economic terms, a one-point increase in the old-age dependency ratio is associated with a 0.0571-unit increase in the standardized embeddedness index. These results indicate that population aging significantly increases the degree of embeddedness of RCEP member states in the medical device trade network, thereby supporting Hypothesis 1.

**Table 5:** Baseline regression results.

Variable	Trade network embeddedness			
	(1)	(2)	(3)	(4)
Old-age dependency ratio	0.0273^**^ (0.0115)	0.0448^***^ (0.0142)	0.0484^***^ (0.0134)	0.0571^***^ (0.0140)
GDP per capita		0.7782^***^ (0.2447)	0.8344^***^ (0.2536)	0.8641^***^ (0.1989)
Population size		−0.2812 (0.5906)	−0.0493 (0.6537)	0.3209 (0.6540)
Urbanization rate		0.0077 (0.0087)	0.0059 (0.0083)	−0.0014 (0.0075)
Trade openness			0.000059 (0.0009)	−0.000090 (0.0008)
Net FDI inflows			−0.0128^*^ (0.0069)	−0.0123^**^ (0.0062)
Internet penetration rate				0.0030 (0.0018)
Labor force participation rate				−0.0004 (0.0085)
Government effectiveness				0.1395 (0.0951)
Controls	NO	YES	YES	YES
Country fixed effects	YES	YES	YES	YES
Year fixed effects	YES	YES	YES	YES
Constant	−0.3713^**^ (0.1565)	−3.2352 (11.1156)	−7.6466 (12.4222)	−14.1868 (12.4306)
*R* ^2^	0.092	0.606	0.631	0.652
Observations	300	300	300	296

Country-clustered robust standard errors are reported in parentheses. ^***^, ^**^, and ^*^ denote significance at the 1%, 5%, and 10% levels, respectively.

### 5.2 Endogeneity tests

The baseline regressions may be subject to omitted-variable bias and reverse causality. On the one hand, difficult-to-observe factors such as healthcare system development, industrial policy, and technical standards may simultaneously affect population aging and trade network embeddedness. On the other hand, countries with a higher degree of trade network embeddedness often have more developed medical industries and public health systems, which may in turn affect population health and age structure. To alleviate these potential endogeneity concerns, the analysis sequentially employs lagged values of the core explanatory variable, includes a lagged dependent variable, and applies an instrumental variables approach.

Table 6 reports the results of the endogeneity tests. First, when the one-period and two-period lags of population aging are used in place of the contemporaneous measure, the coefficients on the core explanatory variable remain significantly positive at the 1% level. Second, after a lagged dependent variable is included in the model, the coefficient on population aging remains significantly positive. Third, the shares of the population aged 50–54 and 55–64 15 years earlier are used as instrumental variables. The first-stage results show that the instruments are significantly correlated with the current old-age dependency ratio. The Kleibergen-Paap rk LM statistic rejects the null hypothesis of underidentification, and the Kleibergen-Paap rk Wald F statistic exceeds 10, suggesting that weak instrument bias is unlikely. The second-stage results continue to show a significantly positive coefficient on population aging, providing further support for the robustness of the baseline findings. The underidentification and weak-instrument diagnostics follow Kleibergen and Paap (48).

**Table 6:** Results of the endogeneity tests.

Variable/test	Trade network embeddedness			Old-age dependency ratio	Trade network embeddedness
	(1) One-period lag	(2) Two-period lag	(3) Dynamic fixed effects	(4) First stage	(5) Second stage
L. Old-age dependency ratio	0.0551^***^ (0.0132)				
L2. Old-age dependency ratio		0.0510^***^ (0.0116)			
Old-age dependency ratio			0.0136^***^ (0.0041)		0.0859^***^ (0.0318)
L. Trade network embeddedness			0.7659^***^ (0.0546)		
Share of population aged 50–54 15 years earlier				0.9322^***^ (0.1914)	
Share of population aged 55–64 15 years earlier				1.1340^***^ (0.2453)	
Controls	YES	YES	YES	YES	YES
Country fixed effects	YES	YES	YES	YES	YES
Year fixed effects	YES	YES	YES	YES	YES
Constant	−13.8296 (13.3093)	−12.0955 (14.1938)	−1.9292 (4.1655)	609.0172^***^ (144.4636)	−36.9213^*^ (22.2757)
*R* ^2^	0.623	0.605	0.837	0.864	0.991
Observations	282	267	282	296	296
Kleibergen-Paap rk LM statistic				43.914	
Kleibergen-Paap rk Wald F statistic				21.957	
First-stage partial *R*^2^				0.369	

Country-clustered robust standard errors are reported in parentheses. ^***^, ^**^, and ^*^ denote significance at the 1%, 5%, and 10% levels, respectively.

### 5.3 Robustness checks

#### 5.3.1 Replacing the dependent variable

Since a single network indicator may not fully capture a country's degree of embeddedness in the medical device trade network, weighted closeness centrality and the weighted clustering coefficient are used separately as alternative dependent variables. As shown in columns (1) and (2) of Table 7, the estimated coefficient on population aging remains significantly positive after the measure of trade network embeddedness is replaced, indicating that the baseline findings are robust.

**Table 7:** Robustness checks using alternative dependent and explanatory variables.

Variable	(1)	(2)	(3)
	Weighted closeness centrality	Weighted clustering coefficient	Share of population aged 65 and above
Population aging	0.0402^*^ (0.0211)	0.0012^***^ (0.0003)	0.0838^**^ (0.0333)
Controls	YES	YES	YES
Country fixed effects	YES	YES	YES
Year fixed effects	YES	YES	YES
Constant	−4.1307 (24.4083)	−0.2474 (0.2681)	−3.9006 (13.4812)
R^2^	0.551	0.590	0.623
Observations	296	296	296

Country-clustered robust standard errors are reported in parentheses. ^***^, ^**^, and ^*^ denote significance at the 1%, 5%, and 10% levels, respectively.

#### 5.3.2 Replacing the core explanatory variable

To mitigate potential measurement error arising from the use of a single aging indicator, the share of the population aged 65 and above is further used in place of the old-age dependency ratio. Column (3) of Table 7 shows that, after replacing the core explanatory variable, population aging continues to have a significantly positive effect on trade network embeddedness in the medical device trade network. This suggests that the main findings are not driven by the choice of aging measure.

#### 5.3.3 Additional robustness checks

As a further robustness assessment, the analysis performs additional checks on the baseline results. (1–2) Adjust the trade volume threshold. The structure of the medical device trade network may be affected by low-volume and sporadic trade relationships. This paper re-constructs the trade network by setting no minimum trade volume threshold and raising the trade volume threshold, respectively, to test whether the conclusion is dependent on specific network construction standards. (3) Exclude the samples from 2020 to 2023. Considering that the demand for medical supplies trade expanded abnormally during the major public health events, and the RCEP came into effect in 2022, which may cause institutional shocks, this paper excludes the samples of this period and re-estimates to eliminate the interference of special period factors. (4–5) Exclude the samples of China and Japan, respectively. China and Japan are economies with large trade scales and strong industrial foundations within the RCEP region, and their network positions may have a significant impact on the overall results. Therefore, the samples of these two countries are excluded, respectively, to test whether the conclusion is driven by a single major country. (6) Apply 1% trimming to continuous variables to reduce the influence of extreme values. (7) Use Driscoll-Kraay standard errors to alleviate the cross-sectional correlation, heteroscedasticity and serial correlation problems that may exist in cross-national panel data. (8) Add country-specific linear trends to exclude the influence of differences in long-term evolution trends among countries. The results in Table 8 show that after the above processing, the estimated coefficient of population aging remains significantly positive, indicating that the core conclusion of this paper has strong robustness.

**Table 8:** Additional robustness checks.

Variable	(1)	(2)	(3)	(4)	(5)	(6)	(7)	(8)
Zero-threshold network embeddedness	USD 1 million threshold	Excluding 2020–2023	Excluding China	Excluding Japan	1% winsorization	Driscoll-Kraay	Country-specific linear trends
Old-age dependency ratio	0.0551^***^ (0.0148)	0.0464^***^ (0.0174)	0.0411^***^ (0.0121)	0.0356^***^ (0.0110)	0.0650^***^ (0.0244)	0.0403^***^ (0.0070)	0.0571^***^ (0.0079)	0.0243^*^ (0.0138)
Controls	YES	YES	YES	YES	YES	YES	YES	YES
Country fixed effects	YES	YES	YES	YES	YES	YES	YES	YES
Year fixed effects	YES	YES	YES	YES	YES	YES	YES	YES
Constant	−12.4902 (14.0576)	−16.7996 (12.0785)	7.7346 (10.1813)	−1.7752 (8.3197)	−17.7282 (14.3248)	−0.9831 (7.9063)	−14.1868 (10.4856)	−1.1260 (11.3803)
*R* ^2^	0.624	0.643	0.637	0.462	0.647	0.623	0.652	0.874
Observations	296	296	239	276	276	296	296	296

Country-clustered robust standard errors are reported in parentheses. ^***^, ^**^, and ^*^ denote significance at the 1%, 5%, and 10% levels, respectively.

### 5.4 Mechanism analysis

To further identify the transmission channels through which population aging affects trade network embeddedness in medical device markets, the analysis examines two mechanisms: export product specialization, and import source consolidation.

Columns (1) and (2) of Table 9 report the results for the export product specialization channel. Column (1) shows that the estimated coefficient on the old-age dependency ratio is −0.0119 in the regression where the logarithm of the number of medical device export product categories is the dependent variable, and the estimate is significant at the 1% level. In column (2), the estimated coefficient on the logarithm of the number of export product categories is −0.4121 and is also significant at the 1% level, indicating that the transmission effect through export product specialization is supported. Economically, the results indicate that population aging narrows the range of medical device products exported and strengthens specialization toward core product categories, thereby increasing trade network embeddedness through stronger trade relationships and closer ties among common trading partners. Therefore, Hypothesis 2 is supported.

**Table 9:** Results of the mechanism analysis.

Variable	Number of export product categories	Trade network embeddedness	Number of import source countries	Trade network embeddedness
	(1)	(2)	(3)	(4)
Old-age dependency ratio	−0.0119^***^ (0.0038)	0.0523^***^ (0.0073)	−3.6374^***^ (0.4827)	0.0478^***^ (0.0065)
Number of export product categories		−0.4121^***^ (0.0940)		
Number of import source countries				−0.0026^***^ (0.0006)
Controls	YES	YES	YES	YES
Country fixed effects	YES	YES	YES	YES
Year fixed effects	YES	YES	YES	YES
Constant	0.8584 (8.3234)	−13.8331^*^ (8.2013)	2715.6805^***^ (456.6067)	−7.2319 (9.1586)
*R* ^2^	0.392	0.697	0.311	0.671
Observations	296	296	296	296
Indirect effect		0.0049		0.0093
Sobel test *p*-value		0.011		0.001

Country-clustered robust standard errors are reported in parentheses. ^***^, ^**^, and ^*^ denote significance at the 1%, 5%, and 10% levels, respectively.

Columns (3) and (4) of Table 9 report the results for the import source consolidation channel. Column (3) shows that the estimated coefficient on the old-age dependency ratio is −3.6374 in the regression where the number of source countries for medical device imports is the dependent variable, and the estimate is significant at the 1% level. In column (4), the estimated coefficient on the number of import source countries is −0.0026 and is also significant at the 1% level, indicating that import source consolidation is an important transmission channel through which population aging increases trade network embeddedness. As population aging deepens, member states become more likely to concentrate import sourcing among a smaller set of source countries. Therefore, Hypothesis 3 is supported.

### 5.5 Heterogeneity analysis

To examine whether the effect of population aging differs across types of economies, the analysis conducts subgroup tests along three dimensions, namely regional characteristics, income level, and stage of population aging, and reports the results of Chow tests. The coefficient-difference tests follow Chow (49).

#### 5.5.1 Heterogeneity between ASEAN and non-ASEAN members

The sample is divided into ASEAN and non-ASEAN groups according to whether an RCEP member state belongs to the Association of Southeast Asian Nations. Columns (1) and (2) of Table 10 show that the estimated coefficient on population aging for non-ASEAN members is 0.0345 and significant at the 1% level, whereas the coefficient for ASEAN members is positive but statistically insignificant. The *p*-value of the Chow test is 0.008, indicating that population aging has a significantly stronger effect on trade network embeddedness in medical device trade among non-ASEAN members. One possible explanation is that the non-ASEAN group includes economies such as China, Japan, South Korea, Australia, and New Zealand, which tend to have larger demand for medical devices, stronger technological foundations, or more advanced population aging. As a result, demographic change in these economies is more likely to translate into stable demand for high-quality procurement and deeper integration into regional supply chains.

**Table 10:** Results of the heterogeneity analysis.

Variable	Non-ASEAN members	ASEAN members	Developing economies	High-income economies	Low-aging economies	Deeply aging economies
	(1)	(2)	(3)	(4)	(5)	(6)
Old-age dependency ratio	0.0345^***^ (0.0020)	0.0173 (0.0272)	0.1156^***^ (0.0229)	0.0211 (0.0211)	0.0228 (0.0292)	0.0586^***^ (0.0075)
Controls	YES	YES	YES	YES	YES	YES
Country fixed effects	YES	YES	YES	YES	YES	YES
Year fixed effects	YES	YES	YES	YES	YES	YES
Constant	13.4551 (18.8244)	−1.0856 (9.5708)	−31.4441^*^ (16.1550)	−1.7402 (10.0901)	34.7963^**^ (15.2487)	−30.9212^***^ (8.7975)
*R* ^2^	0.904	0.410	0.721	0.748	0.544	0.849
Observations	99	197	177	119	137	159
Chow test *p*-value	0.008		0.028		0.057	

Country-clustered robust standard errors are reported in parentheses. ^***^, ^**^, and ^*^ denote significance at the 1%, 5%, and 10% levels, respectively.

#### 5.5.2 Heterogeneity by income level

Based on the World Bank's income classification, the sample is divided into high-income and developing economy groups. Columns (3) and (4) of Table 10 show that the estimated coefficient on population aging for developing economies is 0.1156 and significant at the 1% level, whereas the coefficient for high-income economies is positive but statistically insignificant. The *p*-value of the Chow test is 0.028, indicating that population aging has a stronger marginal effect on trade network embeddedness in medical device trade among developing economies. One possible explanation is that developing economies generally have more limited domestic medical device supply capacity, so the additional demand generated by population aging is more likely to translate into cross-border procurement, greater reliance on external suppliers, and the restructuring of regional supply chain relationships.

#### 5.5.3 Heterogeneity by stage of population aging

Next, the sample is classified into a low-aging group and a deeply aging group based on each member state's average old-age dependency ratio over the sample period, with the median across all member states serving as the cutoff. Columns (5) and (6) of Table 10 show that the estimated coefficient on population aging for the deeply aging group is 0.0586 and significant at the 1% level, whereas the coefficient for the low-aging group is positive but statistically insignificant. The *p*-value of the Chow test is 0.057, indicating that population aging has a more pronounced effect on trade network embeddedness among deeply aging economies. One possible explanation is that deeply aging economies tend to have more stable demand for healthcare, making medical device trade relationships more likely to evolve from general procurement toward sustained, specialized, and relationship-based cooperation.

## 6 Discussion

### 6.1 Relation to existing research

The findings extend the literature on population aging in a direction that has received relatively limited attention. Existing studies have primarily examined how aging affects healthcare expenditure, service utilization, labor supply, and structural transformation (1, 9, 16). The present study adds a cross-border structural dimension by showing that demographic aging is also associated with how countries are positioned within a regional medical device trade network. In particular, the positive effects on weighted closeness centrality and the weighted clustering coefficient indicate that persistent aging-related demand is accompanied by shorter effective trade distances and tighter local relational closure. Thus, the economic consequences of population aging extend beyond changes in the level of healthcare spending to the reorganization of the international supply relationships through which medical devices are obtained and exchanged.

The results also complement the literature on international trade networks. Previous research has emphasized the topology of trade networks, the role of central economies, and the relationship between network position and economic integration (5, 6, 22). By contrast, this study identifies population aging as a demand-side demographic force associated with node-level embeddedness in a specific health-related trade network. This distinction matters because the RCEP medical device network is already nearly saturated, with network density reaching 0.952 by 2023 and the average path length staying close to one. When the extensive margin of bilateral connectivity is close to saturation, additional demographic demand is less likely to be reflected only in the creation of new trade links and more likely to operate through the intensification and stabilization of existing relationships. The positive estimates for weighted closeness and clustering are consistent with this interpretation and with the broader view that repeated transactions can reduce effective trade frictions and strengthen relational ties (32).

The mechanism results further connect several strands of existing research that are usually studied separately. The export specialization channel is consistent with theories of multi-product firms and product-mix adjustment, according to which firms concentrate resources on products with stronger comparative advantages when market conditions and fixed costs make broad product coverage less efficient (36, 37). The import source consolidation channel, in turn, is consistent with relationship-based trade and supplier-selection mechanisms, in which certification, information, switching costs, and service quality increase the value of stable trading relationships (23, 39). Taken together, these findings suggest that population aging does not affect medical device trade through a single demand-expansion channel; instead, it induces coordinated adjustments in demand, export supply, and import sourcing that jointly reshape network structure.

The heterogeneity results qualify these relationships by showing that demographic pressure is not translated into network embeddedness uniformly across economies. The stronger estimates for non-ASEAN members, developing economies, and deeply aging economies suggest that the effect depends on the interaction between demographic demand, domestic industrial capacity, and dependence on cross-border procurement. In more deeply aging economies, healthcare demand is more persistent and therefore more likely to support long-term supplier relationships. In developing economies, relatively limited domestic medical device supply can make additional aging-related demand more dependent on external procurement, increasing the importance of regional trade links. The non-ASEAN result is also consistent with the greater concentration of large markets and advanced medical-device production capabilities in that group. These subgroup findings help explain why similar demographic shifts may generate different trade-network responses across countries.

### 6.2 Theoretical implications

The first theoretical implication is that health capital theory can be extended from the analysis of individual healthcare demand to the study of international trade-network structure. In the conventional health-capital framework, aging accelerates the depreciation of health capital and increases demand for medical services and related products. The evidence in this study suggests a further step: when such demand is persistent, specialized, and supported by repeated procurement, it can alter the relational architecture of cross-border trade. Population aging can therefore be understood not only as a demographic shock to the level of demand, but also as a structural force that changes the intensity, continuity, and configuration of economic relationships among countries supplying health-related products.

Second, the study gives network embeddedness a more explicit economic interpretation by separating regional accessibility from local relational closure. Weighted closeness centrality captures the effective distance between a country and the rest of the regional network, whereas the weighted clustering coefficient captures the density of relationships among its trading partners. Population aging simultaneously improves a country's access to the regional supply system and strengthens the local network connections that support repeated transactions. This two-dimensional interpretation moves beyond conventional bilateral trade measures and shows how a common demand-side force can reshape both the reach and the relational depth of international trade connections.

Third, the two transmission channels imply a demand-supply co-adjustment mechanism. Export product specialization shifts export activity toward a narrower range of product categories, while import source consolidation strengthens repeated transactions with a smaller set of source countries. These adjustments are mutually reinforcing. On the demand side, persistent aging-related demand for medical devices increases the returns to specialization and relationship-specific investment; on the supply side, specialized supply and stable sourcing reduce search, certification, maintenance, and switching costs. In a medical device market characterized by technical standards, after-sales services, consumable replenishment, and regulatory requirements, this feedback process provides a theoretical explanation for why population aging can generate shorter effective trade distances and denser local network ties rather than merely higher trade values.

## 7 Conclusions and policy implications

### 7.1 Conclusions

Using bilateral medical device trade data for the 15 RCEP member states from 2004 to 2023, this study constructs annual directed and weighted trade networks and measures country-level embeddedness by combining weighted closeness centrality and the weighted clustering coefficient. The analysis yields four main conclusions.

First, the regional medical device trade network has evolved toward a highly connected and relatively flat structure. Network density increased from 0.848 in 2010 to 0.952 in 2023, the average clustering coefficient rose from 0.876 to 0.960, and betweenness centralization fell from 0.064 to 0.005. These changes indicate that trade relationships among RCEP members have become increasingly extensive and locally interconnected, while dependence on a small number of intermediary nodes has weakened. At the same time, substantial differences in trade scale and functional position remain, with China, Japan, South Korea, and Singapore occupying important positions and several ASEAN economies becoming more deeply connected to the regional production and distribution system.

Second, population aging is significantly associated with a higher degree of embeddedness in the regional medical device trade network. In the fully specified two-way fixed-effects model, the coefficient on the old-age dependency ratio is 0.0571 and is significant at the 1% level. A one-standard-deviation increase in the old-age dependency ratio corresponds to approximately a 0.56-standard-deviation increase in the embeddedness index, indicating that the estimated relationship is not only statistically significant but also economically meaningful. The result remains positive after a series of robustness tests.

Third, the mechanism analysis shows that population aging affects network embeddedness through coordinated changes in export structure and import sourcing. Aging significantly reduces the number of medical device export product categories, while a narrower export product range is associated with higher embeddedness, indicating that aging promotes greater specialization in medical device exports. In addition, aging significantly reduces the number of import source countries, and a more consolidated sourcing structure is associated with higher embeddedness, indicating that import procurement becomes more concentrated among core source countries.

Fourth, the effect of population aging is heterogeneous across member states. The positive coefficient is statistically significant for non-ASEAN members but not for ASEAN members, and the difference between the two groups is supported by the Chow test. The effect is also substantially stronger among developing economies than among high-income economies, suggesting that aging-related demand in countries with more limited domestic medical device supply is more likely to translate into cross-border procurement and deeper regional dependence. Finally, the effect is significant among deeply aging economies but not among low-aging economies.

### 7.2 Policy implications

Based on these findings, the following policy implications emerge.

RCEP members should establish a demand forecasting and capacity coordination mechanism for aging-related medical devices. Population aging, changes in disease profiles, healthy life expectancy, health insurance coverage, and long-term care needs should be incorporated into the regional framework for industrial cooperation, and medium- to long-term demand lists for key equipment and consumables should be compiled on a regular basis. For aging-sensitive products such as monitoring equipment, imaging devices, dialysis equipment, respiratory support devices, rehabilitation and nursing products, and home-based medical devices, a regional supply-and-demand database could be developed to distinguish replacement demand from demand for new capacity. This would improve the precision of capacity planning, firm investment, and supply chain stress testing.

Regulatory coordination and specialized division of labor in the medical device industry should also be strengthened. Without compromising safety standards, priority should be given to cooperation in registration dossier sharing, electronic certificate verification, and quality system audits for low-risk products with mature standards. For high-risk devices, greater coordination in technical guidelines and post-market surveillance is needed. At the same time, member states should be encouraged to develop specialized products in line with their comparative advantages. Core exporting economies should be guided to move further into key components, software, consumables, and after-sales services, while developing members should be supported in participating in assembly, maintenance, and digital service segments through the cumulative rules of origin.

Supply chain efficiency should be balanced with resilience. Although import source consolidation can reduce the costs of quality verification, regulatory compliance, and after-sales service, it may also increase dependence on a small number of core suppliers. Member states should therefore establish a tiered safeguard mechanism built around “core suppliers plus alternative suppliers” for key equipment, core components, and high-frequency consumables, and conduct supply chain stress tests to identify risks related to common upstream suppliers, single-source components, and critical transport nodes. For developing members, greater efforts are also needed to strengthen trade finance, public procurement information systems, local maintenance capacity, spare parts reserves, and remote maintenance capabilities. Regional supply risks can be further reduced through shared reserves, mutual recognition of emergency authorization, and export coordination.

### 7.3 Limitations and future research

Several limitations should be noted. First, the sample covers the 15 RCEP members over 2004–2023; observations before 2022 characterize trade linkages among these countries retrospectively, and do not identify the causal effect of RCEP implementation. Second, country-level network measures are inherently interdependent within each annual trade matrix. Therefore, the estimates should be interpreted as country-level relationships after controlling for country and year fixed effects and conducting robustness and endogeneity tests. Future research could more clearly disentangle the effects of demographic changes from the institutional effects of RCEP, and further link macro-level trade network positions with product-level dimensions such as export competitiveness, import dependence, and supply chain diversification. This type of cross-sectional dependence is a recognized issue for panel inference when units are exposed to common and interdependent shocks (50).

## Data Availability

The datasets presented in this study can be found in online repositories. The names of the repository/repositories and accession number(s) can be found in the article/supplementary material.
